# Orphan nuclear receptor NR2F6 acts as an essential gatekeeper of Th17 CD4^+^ T cell effector functions

**DOI:** 10.1186/1478-811X-12-38

**Published:** 2014-06-12

**Authors:** Natascha Hermann-Kleiter, Gottfried Baier

**Affiliations:** 1 Department for Pharmacology and Genetics, Translational Cell Genetics, Medical University of Innsbruck, Peter Mayr Str. 1a, A-6020, Innsbruck, Austria

**Keywords:** Autoimmune disease, Pro-inflammatory Th17 cells, Nuclear orphan receptors, Immune response balance and check, NR2F-orphan receptor family, NFAT, AP-1, RORγt, Transcriptional regulation

## Abstract

Members of the evolutionarily conserved family of the chicken ovalbumin upstream promoter transcription factor NR2F/COUP-TF orphan receptors have been implicated in lymphocyte biology, ranging from activation to differentiation and elicitation of immune effector functions. In particular, a CD4^+^ T cell intrinsic and non-redundant function of NR2F6 as a potent and selective repressor of the transcription of the pro-inflammatory cytokines interleukin (Il) 2, interferon y (ifng) and consequently of T helper (Th)17 CD4^+^ T cell-mediated autoimmune disorders has been discovered. NR2F6 serves as an antigen receptor signaling threshold-regulated barrier against autoimmunity where NR2F6 is part of a negative feedback loop that limits inflammatory tissue damage induced by weakly immunogenic antigens such as self-antigens. Under such low affinity antigen receptor stimulation, NR2F6 appears as a prototypical repressor that functions to “lock out” harmful Th17 lineage effector transcription. Mechanistically, only sustained high affinity antigen receptor-induced protein kinase C (PKC)-mediated phosphorylation has been shown to inactivate NR2F6, thereby displacing pre-bound NR2F6 from the DNA and, subsequently, allowing for robust NFAT/AP-1- and RORγt-mediated cytokine transcription. The NR2F6 target gene repertoire thus identifies a general anti-inflammatory gatekeeper role for this orphan receptor. Investigating these signaling pathway(s) will enable a greater knowledge of the genetic, immune, and environmental mechanisms that lead to chronic inflammation and of certain autoimmune disorders in a given individual.

## Lay abstract

Recent research defines nuclear orphan receptor NR2F6 as a critical gatekeeper for Th17-dependent immune effector responses in mouse T cells. Importantly, the ligand binding domain of NR2F6 is evolutionary highly conserved and has been shown by us to be essential for its transcriptional repressor activity, validating NR2F6 as particular druggable target for immune modulation. Thus, this newly defined concept is providing a rational mechanistic basis for a selective agonist of NR2F6 to attenuate the pro-inflammatory cytokines IL-17A, IL-17F, IL-21 and IFNγ production in Th17 cell-mediated immune pathologies. Targeted manipulation of this Th17-subtype selective NR2F6 function may represent a unique therapeutic option to selectively suppress and/or reprogram pathological Th17 cell in i.e. multiple sclerosis patients.

## Introduction

The immune system protects our bodies against invasion by pathogens of viral, bacterial, fungal and parasitic origin and against growth of neoplastic cells. The intensity of the adaptive immune responses must be tightly regulated in terms of class and duration to allow the proper production of immunoregulatory cytokines or chemokines by T lymphocyte subsets and the differentiation of B cells that are able to produce various antibody classes. Subsequently, the interaction between innate and adaptive immune cells provides the best protection for the organism while preventing, as far as possible, collateral damage to bystander tissue. However, because it is a tightrope-walk situation, this delicate balance can be subverted by severe infections, disruption of tissue integrity or genetic susceptibility. Under such conditions, acute immunological pathology such as persistent infection, chronic inflammatory disease and/or autoimmunity may occur.

To be able to modulate such immune diseases clinically, it is essential to both fully understand the positive and negative pathways that regulate immune homeostasis and to predict the adverse effects that could occur during host protection. It is well recognized that such immune cell signaling networks are strongly influenced by an array of distinct nuclear receptors (NRs). In humans the NR super family consists of 48 transcription factors from the steroid hormone, thyroid hormone, oxysterol as well as lipid receptors. In the immune system, NRs are involved in diverse processes such as development, activation, apoptosis, subset differentiation and homeostasis regulation. It is becoming increasingly clear that immune response outputs are orchestrated by both the expression level and the transcriptional activity of several members of the NR family including the glucocorticoid (GR/NR3C1), estrogen (ER/NR3A1&2), vitamin-D3 (VDR3/NR1I1), nuclear receptor subfamily 4, group A, member 2 (NR4A2), peroxisome proliferator-activated receptor γ (PPARγ/NR1C3) or the retinoic acid receptors (RAR/NR1B1). Specifically, these NRs appear to allow a fine tuning of immune cellular processes to environmental changes such as external milieu signals and the cell intrinsic metabolic state during host protection [1-4]. Notably, NRs have been shown to be essential for both the pro-and anti-inflammatory processes in health and disease. Consistently, in both mice and humans, NR mutations have been specifically associated with immune deficiencies or autoimmunity [3,5-9].

The nuclear orphan receptors of the chicken ovalbumin upstream promoter transcription factor (COUP-TF)/NR2F-family are proteins that are involved in a wide range of physiological processes. The NR2F-family consists of three orphan receptors, which are named NR2F1, NR2F2 and NR2F6 in accordance with the Nuclear Receptors Nomenclature Committee (Table 1), and which are central to distinct aspects of metazoan physiology. The NR2F-family members regulate processes as diverse as embryonic and neuronal development, cancer or metabolism.

**Table 1:** **Nomenclature of mammalian NR2F**-**family members and their synonyms (according to**http://www.ncbi.nlm.nih.gov)

**NR2F1**	**NR2F2**	**NR2F6**
ERBA-related protein Ear3	Apolipoprotein regulatory protein 1, ARP1	V-erbA-related protein 2; Ear2
COUP transcription factor I or 2, COUP-TF I or 2	COUP transcription factor II or 2, COUP-TF2, COUP-TFII, Tcfcoup2, COUPTFB	COUP transcription factor 3, COUP-TF3
V-erbA-related protein 3; avian erythroblastic leukemia viral (v-erb-a) oncogene homolog-like 3; Erbal3	ADP-ribosylation factor related protein 1, Aporp1	Avian erythroblastic leukemia viral (v-erb-a) oncogene homolog-like 2, Erbal2

## Review

### Functional domains and transcriptional targets of NR2F-family members

**Figure 1:**
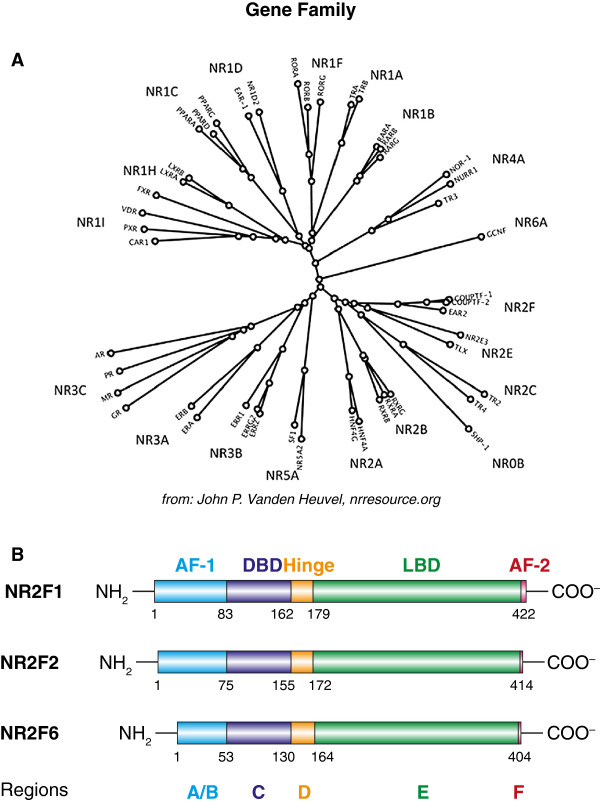
**Schematic protein domain structure of NR2F**-**family members. (A)** Nuclear receptor gene family and its phylogenetic tree (source: nuclear receptor resource; John P. Vanden Heuvel, http://nrresource.org) **(B)** The common domain structures of NR2F1, NR2F2 and NR2F6 from the amino- to the carboxyl-terminus are indicated: the amino-terminal activation function 1 (AF1) domain (also called the A/B region), the central, highly conserved, DNA-binding-domain (DBD) (also called the C region), the hinge region (also called the D region), the ligand-binding domain (LBD) (also called the E region) and the activation function 2 (AF2) domain (also called the F region) and their relative positions. Briefly, the DNA binding domain (DBD), also called C region, of the different mammalian NR2F-family members is highly homologous between different species, which indicated the importance of this critical region for the biological function of these proteins [18]. The A/B region, which is highly variable among different nuclear receptors, is located amino-terminal to the DBD domain. In general, this domain may contain ligand-independent transcriptional activity. The second most conserved region is the ligand binding domain (LBD) or E region, which is responsible for recognition and binding of the receptor’s ligand and for the ligand-dependent transcriptional activity. A relatively short region connects the C region to the E region (region D) and is also known as the hinge domain. Some receptors also contain a region that is carboxy-terminal to the ligand binding domain, which is known as the F region [1,13,18,97].

The family tree and protein domain structure of the NR2F-family that are shown in Figure 1 has been described in detail [10-12]. NR2F-family members homodimerize or heterodimerize with retinoid X receptor (RXR/NR2B1) as well as other nuclear receptors and bind to a variety of response elements that contain imperfect AGGTCA direct or inverted repeats with various spacing on the cognate DNA sequence [13-15]. Although such specific sequences have been described to be preferentially recognized by NR2F-family members, the promoter site-intrinsic features through which distinct NR2F responsive enhancers encode positive versus negative transcriptional outcomes remains unresolved.

Physiological roles of NR2F1 and NR2F2 in non-hematopoietic cells have been reviewed in detail lately and have been identified to be critical regulators in cell differentiation, tissue development, angiogenesis and metabolism [16,17]. The family member NR2F6 was not reviewed by Tsai [12,18,16] although it has been thought to be functionally closely related [15]. However, and unlike Nr2f1 and Nr2f2 knockout mice, Nr2f6-deficient mice are viable and fertile, and they have an underdeveloped *locus coeruleus* of the forebrain, which causes defects in nociception and in circadian clock behavior [19]. Despite its function in the central nervous system NR2F6 is also suggested to repress mouse renin, human oxytocin or rat LH gene transcription [20-24]. Additionally, NR2F6 has been found to be strongly over-expressed in colorectal cancer and to regulate the survival of tumor cells [25].

### Physiological roles of NR2F1, NR2F2 and NR2F6 in hematopoietic cells

The expression of Nr2f1 and Nr2f2 in distinct subpopulations of immune cells has been analyzed [26]. Nr2f1 and Nr2f2 family members are expressed in human CD4^+^, CD8^+^, CD19^+^, and CD14^+^ cells. In addition the expression pattern of the Nr2f gene family has been investigated in resting and activated T, B, NK, and dendritic cells by the ImmGen Consortium (http://www.immgen.org). All Nr2f family members are expressed in diverse adaptive and innate immune cells. Of note, and as a general rule of thumb, the mRNA expression levels of Nr2f6 in hematopoietic cells are increased approximately twofold in comparison to the mRNA expression levels of Nr2f1 and Nr2f2 [27]. Although these Nr2f-family members have been investigated extensively in non-hematopoietic cells, little is known about their critical role in the immune compartment [2,28,29], and as reviewed in [16,18,30]. Possibly this is because Nr2f1 and Nr2f2 deficiencies in mice are fatal and only conditional knockout mice are available for analysis: Owing to the relatively low levels of Nr2f1 and Nr2f2 transcripts in resting lymphocytes [27,31-33], when compared to, e.g., ovary, kidney or brain, such conditional knockouts for the immune compartment have not yet been generated.

However, low levels of expression might also imply that NR2F-family members are powerful regulators of immune cell activation and that expression of this protein family must be tightly controlled in lymphocytes. Because of this possibility and because, dependent upon the microenvironment, memory/effector T cells change expression profiles with their effector differentiation into a specialized subset, it remains a worthy goal to investigate the potential immune system roles of all NR2F-family members in more detail. In fact, signaling pathways that are known to be regulated by NR2F-family members in non-hematopoietic tissues, such as the RAR, TR, VDR, PPARγ, the arylhydrocarbon receptor (AhR), the forkhead box sub-group O (Foxo3a) and the hepatocyte nuclear factor 4 (HNF4/NR2A4) are well known to be critical also in adaptive immune responses [10,12,17,34-38]. NR2F2 generally is thought to functionally interact with many different signaling pathways such as Notch, β-catenin, transforming growth factor-β (TGFβ), HNF4α, runt related transcription factor (Runx)2, PPARγ, CCAAT/enhancer binding protein (C/EBP) α, GATA, RARα or PPARα pathways [34,39-48]. Albeit that all these are known to be highly relevant in inflammatory and/or immune responses, a potential relevance of NR2F2 in specific immune cell subsets has not yet been investigated.

Nevertheless, several studies may indeed indicate a role for all three NR2F-family members in human immune function and in lymphomas and leukemia. Augmented NR2F1 expression has been identified in the pre-disease state of multiple sclerosis patients, potentially indicating a role in the development of autoimmune disease [49]. In the rare autosomal recessive immune disorder, called ICF syndrome (or Immunodeficiency, Centromere instability and Facial anomalies syndrome) microarray experiments and real-time RT-PCR assays revealed significant differences in RNA levels for NR2F2 in lymphoblasts from ICF patients [50]. In humans NR2F2 is strongly upregulated in CD4^+^ and CD8^+^ T cell lymphoma cells, while NR2F6 expression has been shown to be upregulated in lymph nodes [51]. In addition, NR2F6 is significantly upregulated in Anaplastic Large Cell lymphoma compared with Hodgkin lymphoma in human patients [52]. Finally, NR2F6 is deregulated in CD4^+^ T cells of adult T cell leukemia patients [53]. In preclinical leukemia models, NR2F6 has been suggested to regulate the maintenance of the clonogenic status within the cell hierarchy of the cancer cells [54]. While Nr2f6 is highly expressed in hematopoietic stem cells, its expression declines strongly upon normal hematopoietic differentiation as well as the transitions of KSL (c-kit^+^, sca-1^+^, lineage^−^) hematopoietic stem cell to an immature double negative (DN1) T cell stage and from DP to the CD8^+^ SP cells. Of note, Ichim et al., describes a candidate regulatory role of NR2F6 during T cell development. Bone marrow reconstitution experiments with forced overexpression of recombinant NR2F6 resulted in limited T cell development and decrease in thymus size and cellularity [55]. Additionally, NR2F6 expression has also been found to be significantly downregulated in CD19^+^ B cells of systemic lupus erythematosus patients [56].

### NR2F6 appears to restrain Th17-dependent autoimmunity

In addition to its established role in the brain, recent data define NR2F6 as a critical regulatory factor in the adaptive immune system [57] and one of its key functions appears the repression of cytokine production in T cells. The ability of NR2F6 to reversibly suppress the transcription of the TCR/CD28-transactivated transcription factors such as the nuclear factor of activated T cells (NFAT) and the activating protein 1 (AP-1) mechanistically explains its ability to limit IL-2 and IFNγ production. Consistent with this observation, it is well documented that several other NRs, such as the steroid receptors, PPAR, RXR, RAR, and VDR repress the ability of NFAT and/or AP-1 to transcribe their target genes in T cells and this interference is the established basis for the anti-inflammatory actions of corticosteroids [58-63].

Examination of Nr2f6 expression revealed that Nr2f6 mRNA is upregulated in a subset of CD4^+^ T cells that are commonly known as T helper 17 CD4^+^ (Th17) cells [57]. Indeed, in the experimental autoimmune encephalomyelitis (EAE) model, a significantly augmented disease progression of Nr2f6 knockout mice establishes a critical and non-redundant functional threshold mechanism of NR2F6 for repressing Th17 cell pathology both in vivo and in vitro [57]. When biochemically investigating the immune cell intrinsic NR2F6 signaling function(s), NR2F6 appears to counteract the calcium/calcineurin/NFAT-signaling pathway. Mechanistically, NR2F6 directly binds to multiple sites within the Il17a promoter locus and suppresses the DNA accessibility of endogenous NFAT in resting or suboptimally stimulated Th17 cells. In fact, an antagonistic association between NFAT and NR2F6 occupancy on common target genes in T cells strongly suggests a competitive interaction between these TFs. Indeed, NFAT was coimmunoprecipitated with NR2F6 in a DNA scaffold dependent manner, which indicates that these two TFs form a heterodimeric protein:protein complex when bound on DNA. This may either suggest a direct physical competition for high-affinity binding sites and/or subsequently, a transcriptional repression of NFAT by NR2F6. Consistent with this notion, both the physical protein:protein interaction with NFAT and the trans-repression mode of NR2F6 is critically dependent on the DNA-binding and the ligand-binding domains of NR2F6, at least when investigated upon coexpression of recombinant TFs in a T cell line [64].

**Figure 2:**
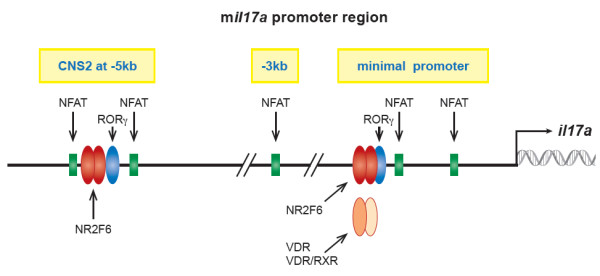
**The *Il17a *promoter and its regulation by orphan nuclear receptors.** The *interleukin (Il) 17a* minimal promoter and conserved non-coding sequences (CNS) are controlled by the general transcriptional machinery and by several nuclear receptors. The DNA consensus binding sequences within the minimal and the CNS2 region of the *Il17a* promoter for NFAT and for nuclear receptors such as NR2F6 and RORγt are shown. Two orphan nuclear receptors, RORγt and NR2F6, thereby have key roles in *Il17a* gene regulation. Of note, NR2F-family members form homo- or hetero-dimers that interact with co-binding partners that are either co-activators or co-repressors. These complexes bind to their consensus sequences (HRE) in the DNA to either inhibit or stimulate target gene transcription. NR2F6 thereby plays a fundamental role in the CD4^+^ T cell compartment by balancing Th17 differentiation and inflammation, as do several other nuclear receptors such as the AHR, RARs, PPARs, LXR, VDR, GR and ER. The lineage-specific master transcription factor RORγt is the only nuclear receptor established to enhance Th17 differentiation and restrains iTregs.

In addition, NR2F6 also directly competes with the Th17 lineage nuclear orphan receptor RORγt for the DNA accessibility to the hormone response elements within the Il17a conserved noncoding sequences (CNS)2 promoter region (Figure 2) [64,65]. Similar to NR2F6, the nuclear receptors RAR, PPAR, LXR, VDR, GR and ER repress Th17 differentiation and protect against the EAE disease mouse model. Nevertheless, the lineage specific TFs that positively induce the differentiation of Th17 cells are RORγt and RORα [66-75]. This opposite behavior of i.e. RORγt might be explained by its DNA binding capability as a monomer whereas all the other NRs (that restrain Th17 cell functions) are described by their DNA binding capabilities as dimers, both as homo- or heterodimers [76,77].

Thus and although these few defined lineage TFs orchestrate synexpression gene clusters that serve in distinct pathways, lineage specificity and plasticity remains a product of the complex combinatorial regulation of gene transcription. Analogous to other pluripotent progenitor cells, particularly naïve CD4^+^ T cells face the challenge of balancing stability and plasticity in their gene expression programs as they differentiate into their highly specialized subsets under the influence of their microenvironment. Recent studies, have generated such a detailed insights into the complexity of T cell lineage differentiation programs especially for the Th17 lineage that appears to be controlled and limited by positive and negative feedback circuits [78,79]. Transcription initiation at sites that are occluded by nucleosomes and high-order chromatin structure is established to require mechanisms for making specific regions accessible to the appropriate regulators [80]. Consistently, chromatin accessibility analysis suggests that such TF complexes pioneer the access of additional TFs and, subsequently, further specify functional subset programming. In the presence of Th17-polarizing cytokines, STAT3, NR2F6 and RORγt are likely to be recruited to many of the same promoters. Intriguingly however, NR2F6 occupancy appears to also strongly overlap with that of NFAT. A long this line of argumentation, such an epigenetic chromatin remodeling role, especially for NFAT2, in transcriptional regulation has been suggested: Because NFAT2 is strongly upregulated in Th0 cells, it has been speculated that the NFAT-mediated pioneering function provides the T cell with plasticity to differentiate in multiple directions, depending on the cytokine environment [81]. Thus, whereas Th17 signals recruit signal-transducer and activator of transcription protein (STAT)3/RORγt to a subset of NFAT/AP-1 binding sites, Th1 or Th2 signals recruit STAT1/T-bet or STAT6/GATA-3 to other NFAT/AP-1 binding sites. Of note, especially Th17 cells are sensitive to low NFAT levels [82], albeit NFAT and AP-1 have also roles in other Th subsets [83]. Consistently, NR2F6 may serve as an integrated regulator of the defined Th17 helper cell lineage identity and/or plasticity that functions by repressing transcriptional programs at key gene loci.

**Figure 3:**
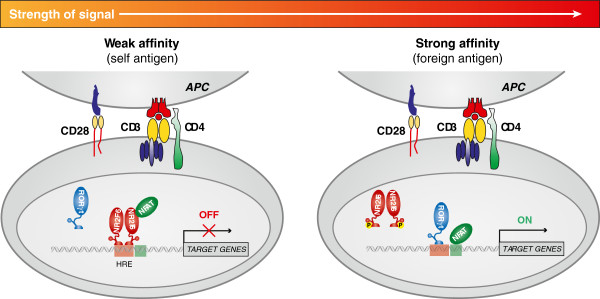
**Nuclear receptor NR2F6**, **as a signal**-**induced functional switch**, **that safeguards Th17 cell effector functions.** A schematic representation of signaling events in Th17 cells is shown: NR2F6 serves as a TCR/CD28 signaling threshold regulated barrier against autoimmunity where NR2F6 is part of a negative feedback loop that limits inflammatory tissue damage. In the absence of a strong antigenic signal, NR2F6 remains pre-bound to the signal response element (RE) and recruits co-repressors to suppress transcription of defined gene loci. In high-affinity antigen-stimulated T cells, however, NR2F6 is phosphorylated by PKC and, thereby, loses its DNA binding capacity; subsequently, NFAT and/or RORγt are able to bind to their promoter/enhancer sequences and recruit additional co-activators to activate transcription of, e.g., *Il17a*. In robustly activated CD4^+^ T cells, NFAT and AP-1 bind cooperatively to sites throughout the genome. Thus and intriguingly, NR2F6 emerges as a prototypical repressor that, in the absence of high affinity antigen receptor stimulation, “locks out” Th17 lineage programming. Because Th17 cell-intrinsic function of NR2F6 renders Th17 cells less pathogenic by repressing key cytokine transcription, NR2F6 agonists may provide a rational basis for the treatment of Th17-mediated autoimmune pathologies.

Importantly, and in contrast to other NRs, constitutively lymphocyte-expressed NR2F6 is already prebound to its hormone response elements within i.e. the Il17a cytokine promoter loci in a resting state and thereby may simply inhibit the induced DNA binding capacities of activation-dependent TFs (see for our working model cartoon in Figure 3). The current experimental evidence thus might suggest that NR2F6 similarly affects responses by remodeling the chromatin landscape, which would critically control the subsequent recruitment of other TFs involved in regulating the expression of adjacent genes. Next to IL-17, Nr2f6-deficient T cells also repress IL-2 and IFNγ, which indicate that NR2F6 may serve as a more general gatekeeper by directly preoccupying binding sites for NFAT to prevent transcriptional activation [57]. This conveys an important message for Th17 subset selectivity and indicates that NR2F6 specifically counteracts the generation of the highly pathogenic Th17 cell type and decreases the risk of promoting autoimmunity [57]. Intriguingly and of note, NR2F6 however appears to alter the transcription levels of only a selected subset of genes rather than promote large-scale changes in gene expression. The exact mode of action of NR2F6 function, however, remains further detailed investigations. In contrast to Th17 cells, NR2F6 appears dispensable, or at least less critical, for Th1 and Th2 functions. This renders NR2F6 an exceptional drug target because therapeutic intervention would not be expected to perturb the generic regulatory programs that are shared by other immune cell types. In this context, it will be necessary to compare the global distributions of NR2F6 and NFAT and of lineage-specifying TFs in Th17, iTreg, Th1 and Th2 cells. NR2F6 might thereby appear as a prototypical repressor candidate that functions to specifically antagonizes Th17 lineage programs.

### NR2F6 is itself regulated

### Nr2f6 mRNA

Because CD4^+^ T lymphocytes can differentiate into several diverse subsets depending on microenvironmental milieu factors and similarly to the FOXO transcription factor family, expression of the NR2F-family might influences cellular potential [84]. We showed that the expression of NR2F6 was regulated upon TCR/CD28 stimulation and cytokines in the Th17 polarization milieu strongly increased Nr2f6 expression in T cells [57]. Thus, transcriptional regulators of the Nr2f6 locus, albeit currently undefined, appear to be induced in response to Th17-polarizing conditions. In silico analysis strongly indicates, that the Nr2f6 promoter may have functional STAT and NR sites [85].

### Post-translational modification (PTM) of NR2F6

Antigen receptor-induced protein kinase (PK)C-mediated phosphorylation has been shown to inactivate NR2F6, thereby allowing for robust cytokine responses [57]. Mechanistically, Ser-83, which is located within the DBD of NR2F6, appears to be the main phosphorylation site upon TCR stimulation to abrogate the DNA-binding capacities of NR2F6 and subsequently allows NFAT/AP-1 binding at the given promoters. Thus, only phosphorylation of NR2F6, which is mediated by a sustained PKC activity downstream of full and robust TCR/CD28 activation, promotes unopposed NFAT and/or RORγt-mediated DNA binding at the critical cytokine gene loci such as Il2 and Il17a. Similarly, IL-2 secretion is established to be inhibited by several other NRs, such as the ER (NR3A), RAR (NR1B), PPARs (NR1C), LXR (NR1H3) VDR (NR1I1), and GR (NR3C1) [61,86-90], whereas AhR activates the Il2 promoter [91]. Similarly to NR2F6, the DNA binding domains of other nuclear receptors, such as Nur77 (NR4A1), RARα (NR1B1), ERα (NR3A1), or the VDR (NR1I1), are directly phosphorylated by PKC or PKA as reviewed previously [92,93]. A significant difference between the formerly mentioned NRs and NR2F6 is the constitutive presence of NR2F6 at its hormone response element (HRE) of the DNA in resting cells, whereas the other NRs are activated via environmental stimuli and subsequently adapt the cell to the microenvironment through their DNA binding capability. Additionally, the number and diversity of other PTM covalent modifications of NR2F6, such as acetylation, ubiquitination or sumoylation which are well known to shape nuclear receptor activity [9,94], have just begun to be analyzed.

### NR2F6-Ligand?

Although the NR2F-family remains defined as orphan NRs, a significant interest in the identification of the detailed molecular mechanisms and identification of endogenous ligands that may regulate these NRs exists. Of note, a recent study has shown that NR2F2 is a retinoic acid-activated receptor [95]. This ligand-regulated feature of NR2F2 (albeit retinoic acid has a rather low affinity) has left open the possibility that, although currently undefined, endogenous NR2F-family specific ligands may exist as agonists or antagonists and may modulate the functions of NR2F-family members. In this regard, the findings that high levels of retinoic acid trigger iTreg formation might be relevant [95]. Detailed studies are now urgently needed to determine the physiological consequences of mRNA regulation, covalent PTM modifications, and the existence of modulatory function(s) of endogenous ligands of NR2F6 function in detail.

### NR2F6 as a drug target in molecular medicine

Environmental factors and gender undoubtedly play key roles in the susceptibility to autoimmune diseases, but clearly genetic disposition is also important. These NR2F6–mediated transrepression is proposed to have roles in controlling both the initiation, magnitude and duration of pro-inflammatory gene expression, and, thus, in targeting NR2F6-specific mechanisms, both local and systemic inflammation appear amenable to clinical manipulation. NR2F6 could potentially represent such a genetic risk factor for the development of autoimmune diseases through its regulation of pathogenic Th17 cells. In deed, NR2F6 has been identified as a type 1 diabetes susceptible SNP (http://arxiv.org/pdf/1404.4482.pdf) and as a risk allele in autoimmune leukocytes [96]. It now will be interesting to further validate this potential genetic association of allelic NR2F-family variants/SNPs with health and disease.

Of note, nuclear receptors have been a rich source of drug targets, especially for inflammation-mediated diseases but also in lipid, carbohydrate and energy homeostasis [32]. Over the past few years, significant breakthroughs in the identification of ligands both from natural as and from synthetic sources have occurred, as reviewed by Burris [97]. The newly defined repressor, NR2F6, may be a compelling and well-validated new target for regulating the Th17 lineage balance and for switching pathogenic Th17 into non-pathogenic cells. Furthermore, the effect would be largely specific for Th17 cells because we did observe comparable effector response outcomes during the differentiation of the other CD4^+^ T cell subset. In addition, the lack of maintenance function of NR2F6 in adult organs renders it a potentially safe target for the treatment of immune diseases.

Taken together, this NR2F6-centered pathway identified may very well offer new targets that are aimed at blocking the generation of pathogenic Th17 cells for the treatment of autoimmune diseases. Although our current preclinical knowledge strengthens this hypothesis, the direct causality of NR2F6 and incidence of human autoimmune disease remains yet to be demonstrated (Table 2).

**Table 2:** Physiological functions of NR2F family members and their roles in disease

	**NR2F1**	**NR2F2**	**NR2F6**
**Immune system**	**Autoimmunity, expressed CD4**^**+**^**/CD8**^**+ **^**and CD19**^**+ **^**cells **[26,49]	**Autoimmunity, expressed CD4**^**+**^**/CD8**^**+ **^**and CD19**^**+ **^**cells **[26,50,51]	**T cell development, activation thresholds, CD4**^**+ **^**Th17 autoimmunity, leukemia**[51,53,54,57,64,98]
**T cells**[27], http://www.immgen.org	+	+	+++
**B cells**[27], http://www.immgen.org	+	+	+++
**Development**	Embryo, eye, hair, ear, angiogenesis [99]	ES cell differentiation, mesenchyme, angiogenesis, vessel identity, cerebellum, lymphangiogenesis, stomach, eye, osteoblast [34,100-102]	
**Hormone regulation**	LHβ, Vegf, LH, receptor, RAR [103]		LH receptor, oxytocin [22-24,104]
**Cancer**		Breast & prostate cancer, MC-lymphoma, metastasis, AML [43,105]	Colorectal, survival [25,52,54,106]
**Metabolism**	Lipid uptake [41,103]	Insulin secretion, adipogenesis, energy metabolism	Lipid metabolism [107,108]
**CNS**	Axons formation	[109]	Circadian rhythm, nociception [19,110-112]
	Cortex patterning		
	Fate specification		
**Kidney**		Renin production	Renin production [20,21,113]
**Reproduction**		Reproduction	

The expression pattern of the Nr2f gene family has been investigated in resting and activated T and B cells by the ImmGen Consortium (http://www.immgen.org). All Nr2f family members are expressed in diverse adaptive and innate immune cells. Of note, and as a general rule of thumb, the mRNA expression levels of Nr2f6 in hematopoietic cells are increased approximately twofold in comparison to the mRNA expression levels of Nr2f1 and Nr2f2 dipicted as + versus +++. Nr2f1 and Nr2f2 in non-hematopoietic tissues were reviewed recently [16], therefore, only new and immune relevant references, as well as those for Nr2f6, are shown.

## Conclusions

Here, we describe recent studies that extend our understanding of how the NR2F-family member NR2F6 exerts important regulatory roles in adaptive immunity. In our model, NR2F6 exerts its Th17-negative regulatory function as a transcriptional repressor that competes with Th17-positive transcription factors over binding sites, which is analogous to the action of other NRs. Importantly, however, NR2F6 appears to alter the transcription levels of only a very selected subset of genes rather than promoting large-scale changes in gene expression. Of note, NR2F6 may act differently on various promoters to modulate transcription, and its effect may be dependent upon epigentics or possibly concurrent direct and indirect interactions between other proteins and NR2F6. NR2F6 might also share functions with its paralogs NR2F1 and NR2F2 in the repression of key cytokine mRNAs that control Th17 cells and systemic inflammation. Studies that include genome-wide approaches and detailed analyses of NR2F-family-dependent genetic programs will be essential to comprehensively understand the relationship between NR2F-family activity and distinct human diseases.

Nevertheless, our current functional model provides an excellent starting point for deciphering the underlying physical interactions with DNA binding profiles or protein–protein interactions. Specifically, mechanistic studies will be able to determine the physiological consequences of transcriptional regulation and the covalent modifications of NR2F6 in detail. Because nuclear receptors are well-established drug targets, pharmacological modulation of NR2F6 may represent an innovative therapeutic regimen for counteracting the pathogenic phenotype of Th17 cells.

### Non-standard abbreviations used

AhR, arylhydrocarbon receptor; AICD, activation induced cell death; AP-1, activating protein-1; Bcl6, B-cell lymphoma 6 protein; BMAL1/ARNTL, aryl hydrocarbon receptor nuclear translocator-like; C/EBPα, CCAAT/enhancer binding protein; ChIP, chromatin immunoprecipitation; CNS, conserved noncoding sequences; Clock, circadian locomoter output cycles kaput; DBD, DNA binding domain; EAE, experimental autoimmune encephalomyelitis; ER/NR3A, estrogen receptor; Foxo3a, the forkhead box sub-group O; GATA3, GATA binding protein 3; GM-CSF, granulocyte macrophage-colony stimulating factor; GR/NR3C1, glucocorticoid receptor; HNF4/NR2A4, hepatocyte nuclear factor 4; HRE, hormone response element; IFN, interferon; IL, interleukin; LBD, ligand binding domain; LXR, liver X receptor; N-CoR, nuclear receptor co-repressor; NFAT, nuclear factor of activated T cells; NFκB, nuclear factor of κB; NR, nuclear receptor; NR4A2, nuclear receptor subfamily 4, group A, member 2; NR2F/COUP-TF, chicken ovalbumin upstream promoter transcription factor; PK, protein kinase; PPARγ/NR1C3, peroxisome proliferator activated receptor; RAR/NR1B1, retinoic acid receptor; REV-ERBα/ NR1D2, V-erbA-related protein 1-related; RORγt, retinoid-related orphan receptor-gamma; Runx, runt related transcription factor; RXR/NR2B1, retinoid X receptor; SMRT, silencing mediator for retinoid and thyroid hormone receptors; STAT, signal-transducer and activator of transcription protein; T-bet, T-box transcription factor TBX21; TCR, T cell receptor; TF, transcription factor; Tfh, follicular helper T cells; TGFβ, transforming growth factor-β; Th, T helper; TNF, tumor necrosis factor; TR, thyroid hormone receptor; VDR3/NR1I1, vitamin-D3 receptor; XRE, xenobiotic response elements.

## Competing interests

The authors declare that they have no competing interests.

## Authors’ contributions

Both authors contributed equally to this review article. Both authors read and approved the final manuscript.
